# Effect of farrowing pen size on pre-weaning performance of piglets

**DOI:** 10.1093/tas/txab123

**Published:** 2021-07-19

**Authors:** Katherine D Vande Pol, Austin L Laudwig, Aaron M Gaines, Beau A Peterson, Caleb M Shull, Michael Ellis

**Affiliations:** 1 Department of Animal Science, University of Illinois, Urbana-Champaign, IL 61801, USA; 2 The Maschhoffs LLC, Carlyle, IL 62231, USA

**Keywords:** farrowing pen size, piglet, pre-weaning mortality, weaning weight

## Abstract

Litter sizes in commercial pig production have increased substantially over recent years; however, farrowing pen sizes have generally not changed over the same time period. The objective of this study was to evaluate the effect of farrowing pen size on piglet pre-weaning growth and mortality. Differences in pen size were created by varying the width of pens of the same length, increasing the creep area available to the piglets. The study used a total of 1,786 litters in a randomized complete block design to compare two farrowing pen size treatments (FPS): Standard (pen width 1.52 m) and Increased (pen width 1.68 m). Litter sizes were equalized across treatments (12.9 ± 1.95 piglets) at 24 h after birth using cross-fostering. Litter weights were collected at birth and weaning (21.3 ± 2.08 d); pre-weaning mortality was recorded. The experimental unit was the litter; models for statistical analysis included FPS and replicate. Farrowing pen size had no effect (*P* > 0.05) on litter size at birth (12.8 and 13.0 for the Standard and Increased FPS, respectively), after cross-fostering (12.9 for both treatments), or at weaning (11.2 and 11.3, respectively). There was no effect (*P* > 0.05) of FPS on total litter or average piglet weight at birth, after cross-fostering, and at weaning. These results suggest no benefit in piglet performance from increasing the width of farrowing pens. As litter sizes continue to increase in commercial production, further research is warranted to re-evaluate the impact of farrowing pen size on pre-weaning mortality.

## INTRODUCTION

Substantial increases in litter size have occurred in commercial swine production over recent years which have been accompanied by increases in pre-weaning piglet mortality. The number of piglets born alive to sows on U.S. units currently averages approximately 13.5; this number has increased by approximately three piglets over recent years (PigChamp, 2004, 2020). Pre-weaning mortality levels have also increased over this time period and currently average in excess of 15% (PigChamp, 2004, 2020). Despite this increase in the number of piglets per litter, farrowing pen sizes used on commercial operations have generally not changed and are based on historical recommendations (e.g., Midwest Plan Service, 1991). In addition, the primary cause of piglet pre-weaning mortality under commercial conditions continues to be crushing by the sow (Vande Pol et al., 2021a, b). Conceptually, increasing the size of farrowing pens could reduce the risk of piglets being crushed by providing more space to avoid the sow. However, there has been limited controlled research to evaluate this concept. Most studies that have directly compared farrowing pen size used loose-housed sows (Cronin et al., 1996; Weber et al., 2009; Grimberg-Henrici et al., 2019). However, most U.S. facilities utilize farrowing crates to confine sows during lactation (McGlone, 2013). Only one large-scale controlled study has compared farrowing pens of differing sizes in facilities which used sow crates; however, this study found no effects of pen size on piglet mortality or growth (Leonard et al., 2020). Further research is necessary to determine the effects of farrowing pen size on piglet pre-weaning performance using the large litter sizes that are typical of current commercial production.

## MATERIALS AND METHODS

This study was carried out on a commercial sow facility of The Maschhoffs, LLC, located near Pittsfield, IL. Protocols for this study were approved by the University of Illinois Institute of Animal Care and Use Committee prior to the start of the research.

### Animals, Facilities, and Management

This study was carried out in a standard commercial farrowing facility (with individual pens and sow crates) from day 112 of gestation (when sows were moved into the farrowing facilities) until weaning (21.3 ± 2.08 d after farrowing). Sows were from commercial crossbred lines and had been mated to commercial sire lines; sow parities ranged from 1 to 6. Housing and management of sows and piglets were in line with commercial procedures and practices. Four farrowing rooms were utilized, two of which (Rooms 1 and 2) contained 24 individual farrowing pens; the other two (Rooms 3 and 4) contained 26 individual farrowing pens. Each pen had solid side walls and perforated plastic flooring, a farrowing crate, and one heat lamp suspended over a rubber mat in the center of one side of the pen (creep area). An additional heat lamp, suspended on the opposite side of the pen, was provided during the cooler months (November to May) for the first few days after farrowing.

The thermostat to control ambient temperature in each room was set at 22.5°C on the day of farrowing, then gradually reduced to 19°C by d 15 after farrowing, remaining at this temperature until weaning. Room temperature was maintained using heaters, evaporative cooling cells, and fan ventilation as needed. All sows that had not farrowed by d 114 of gestation were induced on d 114 to farrow on d 115 using 2 cc of prostaglandin F2α (given at 0600 h; Lutalyse, Pfizer Animal Health US).

During gestation and lactation, sows were fed diets formulated to meet or exceed the nutritional requirements proposed by the National Research Council (2012). During gestation, sows were housed in either individual crates or in pens with groups of 8 sows. From entry into the farrowing room until farrowing, sows were fed 1 kg of feed twice each day (at 0600 h and 1400 h). Subsequently, sows had *ad libitum* access to feed throughout lactation via a sow-operated feed dispenser attached to the feed trough. Sows and piglets had *ad libitum* access to water via cup-type drinkers located next to the sow feeding trough and in the farrowing pen, respectively. Piglet cross-fostering was carried out at 24 h after birth such that litter sizes were equalized across treatments. Standard piglet processing tasks (including tail docking, physical castration of males, and iron and antibiotic injections) were carried out at 5 days after birth.

### Experimental Design and Treatments

The study used 1,786 litters in a randomized complete block design; sows within a block were from the same type of gestation housing system, were housed within the same area of the same farrowing room, had the same insemination date, and similar parity (± 1) and body condition score at farrowing (± 1). Sow genetic line was balanced across treatments over the study period. Body condition score was based on a 5-point scale (1 = extremely thin to 5 = extremely fat); parity was defined as the number of litters born including that used in this study. Two farrowing pen size treatments were compared: Standard (farrowing pen width 1.52 m); Increased (farrowing pen width 1.68 m). Farrowing pen and crate dimensions are presented in Table 1. For the Standard treatment, the farrowing crate was located in the center of the pen; the crate was 0.55 m wide, resulting in a distance between the side of the crate and the pen wall of 0.49 m on each side of the crate. For the Increased treatment, the extra 0.16 m pen width was on the side of the pen with the heat lamp resulting in distances between the side of the crate and the pen wall of 0.49 m and 0.64 m. Farrowing pen and crate lengths differed by room and widths differed according to treatment, which resulted in differences between rooms for the floor area within the pen and the crate (Table 1).

**Table 1.** Farrowing pen and crate dimensions by farrowing pen size treatment and room

Treatment^1^	Rooms	Pen width, m	Pen length, m	Pen area, m^2^	Crate width, m	Crate length, m	Crate area, m^2^
Standard	1 and 2	1.52	2.04	3.10	0.55	1.95	1.07
Standard	3 and 4	1.52	2.19	3.33	0.55	2.10	1.16
Increased	1 and 2	1.68	2.04	3.43	0.55	1.95	1.07
Increased	3 and 4	1.68	2.19	3.68	0.55	2.10	1.16

^1^Standard = pen width of 1.52 m. Increased = pen width of 1.67 m.

### Procedures and Measurements

The date of farrowing and the number of piglets born alive, stillborn, and mummified for each sow was recorded and used to calculate the total number of piglets born per litter (alive and stillborn). After farrowing was complete, all piglets born alive were weighed as a group to obtain total born alive weights and calculate average piglet birth weight. All stillborn piglets were weighed as a group to obtain total stillborn weights and calculate average stillborn piglet weight. Total litter weight was calculated as the sum of the total weights of piglets born alive and stillborn. Litters were weighed as a group at weaning to obtain a total litter weaning weight, which was used to calculate average piglet weaning weight. Weigh scales (Digi-Star model SW4600EID scale; Digi-Star LLC, Fort Atkinson, WI; accurate to 0.2 kg) for measurement of litter birth and weaning weights were validated prior to each use with standard check weights that approximated to the average expected total litter birth and weaning weight (i.e., 25.0 and 50.0 kg, respectively). Litters were checked daily and the dates and causes of piglet deaths were recorded.

### Statistical Analysis

All data were analyzed using SAS v. 9.4 (SAS Inst. Inc., Cary, NC). The PROC UNIVARIATE procedure of SAS was used to verify normality and homogeneity of variances of the residuals. All variables that conformed to the assumptions of normality and homogeneity were analyzed using the PROC MIXED procedure of SAS (Littell et al., 1996); all other data were analyzed using PROC GLIMMIX. The experimental unit was the sow and litter for all measurements, and the model accounted for the fixed effects of farrowing pen size treatment and random effects of block, parity, and the interaction between farrowing pen size and parity. Least-squares means were separated using the PDIFF option of SAS, being considered different at *P* ≤ 0.05. All *P*-values were adjusted using a Tukey’s adjustment for multiple comparisons.

## RESULTS AND DISCUSSION

Least-squares means for treatment differences in sow parameters and effects on litter performance are presented in Table 2. Sow parameters (i.e., parity, body condition score, and total teat number) were similar (*P* > 0.05) for the two treatments. Body condition scores and teat numbers were comparable to those reported for contemporary commercial sow populations (Maes et al., 2004; Kim et al., 2005; Vande Pol et al., 2021a, b). The average parity of sows used in the current study was relatively low (2.7) compared to that typically observed in commercial herds (Maes et al., 2004; Vande Pol et al., 2021a, b). The unit used for this study was repopulated with first litter gilts at the start of the study, resulting in a relatively greater proportion of sows with low parities than would typically be observed.

**Table 2.** Least-squares means for the effect of farrowing pen size treatment on sow parameters and litter performance

	Farrowing pen size^1^			
Item.	Standard	Increased	SEM	*P*-value
Number of litters	897	889	-	-
Total number of piglets (born alive)	11485	11576	-	-
Sow parameters				
Parity^2^^,^^3^	2.68	2.69	-	0.97
Body condition score^2^^,^^3^	3.14	3.14	-	0.92
Total teat number	15.68	15.63	0.064	0.37
Number of piglets				
Born alive	12.80	13.02	0.184	0.18
Stillborn^2^	0.77	0.80	-	0.48
Mummified^2^	0.36	0.34	-	0.52
Total born^5^	13.57	13.83	0.184	0.12
After cross-fostering	12.88	12.94	0.115	0.55
Weaned	11.20	11.29	0.124	0.4
Litter birth weight, kg				
Born alive	17.56	17.99	0.289	0.07
Stillborn^2^	0.82	0.88	-	0.5
Total born^5^	18.37	18.87	0.293	0.05
Average piglet birth weight, kg				
Born alive	1.55	1.55	0.014	0.91
Stillborn	1.25	1.25	0.034	0.86
Total born^5^	1.53	1.53	0.014	0.76
Weaning weight, kg				
Litter	74.18	75.50	1.414	0.11
Average piglet	6.81	6.83	0.075	0.74
Pre-weaning mortality^2^				
Within 24 h of birth, % of piglets born alive	1.7	1.7	-	0.97
After cross-fostering, % of piglets after cross-fostering	11.3	11.1	-	0.79
Total, % of piglets born alive	12.9	12.5	-	0.40
Total, % of piglets after cross-fostering	13.0	12.7	-	0.54
Cause of mortality, % of piglets that died pre-weaning^2^				
Crushed	59.2	56.4	-	0.16
Starved	4.3	4.5	-	0.83
Low viability	14.9	15.1	-	0.89
Other	21.6	24.0	-	0.16
Piglet age at death, d^2^	4.9	5.4	-	0.11

¹Standard = pen width of 1.52 m. Increased = pen width of 1.67 m.

²Data did not meet assumptions of normality and were analyzed using Proc Glimmix of SAS.

3Parity = total number of litters including the one used in the study.

4On a 5-point scale; 1 = extremely thin, 5 = extremely fat.

5Piglets born alive and stillborn.

There was no effect (*P* > 0.05) of farrowing pen size treatment on either the number of piglets at birth (born alive, stillborn, mummified, and total born) or on the total litter or average piglet weight at birth (Table 2). These results were expected, as these measurements were taken before the treatments would be anticipated to have impacted piglet performance. In addition, litter size after cross-fostering was similar (*P* > 0.05) for the two treatments which was the objective of the cross-fostering procedure. Litter sizes and piglet birth weights were similar to those reported by other recent studies carried out on commercial farms (Feldspausch et al., 2019; Vande Pol et al., 2020a, b, 2021c).

Farrowing pen size had no effect (*P* > 0.05) on either number of piglets weaned or litter and average piglet weights at weaning. Piglet mortality within the first 24 h of birth and after cross-fostering, and total pre-weaning mortality were not different (*P* > 0.05) between treatments (Table 2). In addition, there was no effect (*P* > 0.05) of treatment on either the causes of mortality, or the average age of piglets at death (Table 2). These results indicate that there was no benefit from increasing farrowing pen size for piglet performance. A factor that could influence the effect of farrowing pen size is the parity number of the sows, which was relatively low in the current study (2.7) compared to that commonly observed on commercial farms (Maes et al., 2004; Vande Pol et al., 2021a, b). Litter sizes at birth generally increase with parity up to approximately the fifth parity (Lavery et al., 2018). This was the case in the current study, with the number of piglets born alive increasing quadratically (*P* < 0.05) with parity (11.5, 12.8, 13.8, 14.1, 13.1, and 13.0 piglets for parities 1, 2, 3, 4, 5, and 6, respectively). It might be expected that increasing farrowing pen size could have a greater effect in larger litters. However, in the current study, the average litter size at birth after cross-fostering (12.8 to 13.0; Table 2) was close to values reported for number of piglets born alive for U.S. commercial herds (PigChamp, 2020). In addition, there was no interaction (*P* > 0.05) between farrowing pen size treatment and parity for any of the piglet performance measures (data not reported), suggesting that the Increased treatment was not effective, even for larger litters.

The sizes of farrowing pens widely used by the U.S. commercial industry are generally based on outdated historical recommendations. For example, Midwest Plan Service (1991), a source that is commonly used by the industry, recommends farrowing pen width and length of 1.52 m and 2.13 m, respectively, dimensions that are similar to the Standard treatment of the current study. The widths of the Increased treatment pens were between 10% and 12% greater than those of the Standard treatment, depending on farrowing room (Table 1), with the extra space being on the side of the pen where the heat lamp was located. This is commonly considered as the “creep” area and is designed to attract the piglets to a part of the pen away from the sow to reduce mortalities resulting from crushing (Larsen et al., 2017). There have been a number of attempts to define the optimum size of the total creep area required for a litter based on piglet body measurements. Estimates have varied from 0.56 m^2^ (for a litter of 12 piglets up to 18 d of age; Zhang and Xin, 2005) to 1.7 m^2^ (for 10 piglets at 21 d of age; Vasdal, 2007). For piglets weaned at 21 d of age, a creep area of 0.9 m^2^ for litters of 14 piglets was recommended by Fels et al. (2016) and, also, by Wheeler et al. (2007) for litters of 10 piglets. Similarly, Meyer et al. (2012) recommended 0.72 to 1.1 m^2^ for litter sizes of up to 12 piglets weaned at 28 d of age. In the current study, the area of the pen on the side with the heat lamp was between 1.0 and 1.2 m^2^ for the Standard treatment and 1.4 and 1.5 m^2^ for the Increased treatment (Table 1). These areas are within the range of estimates discussed above.

The highest levels of pre-weaning mortality generally occur in the first few days after birth (Su et al., 2007) with the primary cause being due to crushing by the sow (Dyck and Swierstra, 1987; Vande Pol et al., 2021a, b). The current study was based on the premise that increasing the size of the farrowing pen would increase the distance between the piglets and the sow, thereby reducing the likelihood of piglets being crushed. However, piglets are often attracted to the sow in the early period after birth despite the presence of a localized heat source in the farrowing pen intended to draw them away (Houbak et al., 2006; Pedersen et al., 2006). In addition, the increased space was only on one side of the farrowing pen, and piglets could still be close to the sow on the narrower side of the pen. Further research is required to understand the effect of farrowing pen size on piglet behavior, particularly during the early postnatal period.

Most previous research on farrowing pen size has been carried out with sows that were loose-housed rather than confined in crates. Systems based on farrowing pens with loose-housed sows commonly have greater total pen floor space, but usually have higher pre-weaning mortality and/or lower weaning weights than those using crates (Robertson et al., 1966; Marchant et al., 2000; Bates et al., 2003; Pedersen et al., 2011; Kobek-Kjeldager et al., 2020). However, studies have shown that temporarily confining sows in crates for the first few days after farrowing results in pre-weaning mortality levels similar to systems where sows are confined throughout lactation (Mousten et al., 2013; Chidgey et al., 2015). This suggests that differences in pre-weaning mortality between loose and crated farrowing systems are likely due, in part at least, to differences in sow behavior rather than pen floor area *per se*.

Studies that have evaluated effects of farrowing pen size with loose-housed sows have produced conflicting results. Cronin et al. (1996) found no effect of increasing farrowing pen area from 3.4 to 4.3 m^2^ on piglet growth or mortality. Similarly, Weber et al. (2009) evaluated risk factors across farrowing systems using commercial farm records and found no significant effect of the size of the farrowing pen on piglet mortality for pen areas ranging from 5.1 to 8.6 m^2^. In contrast, Grimberg-Henrici et al. (2019) reported that increasing farrowing pen area from 5.2 m^2^ vs. 6.2 m^2^ reduced pre-weaning mortality and the percentage of piglets that were crushed.

Only one published study has compared farrowing pen sizes utilizing sows kept in crates during lactation. Leonard et al. (2020) found no effect on piglet pre-weaning mortality or weaning weight from keeping sows and litters during lactation in pens with floor areas of either 3.2 m^2^ (pen dimensions 1.52 by 2.13 m) or 4.5 m^2^ (pen dimensions 1.83 by 2.44 m), results that are similar to those of the current study. The dimensions and area of the smaller pens in the study of Leonard et al. (2020) were similar to the Standard treatment of the current study (Table 1). However, the larger pen size in the study of Leonard et al. (2020) was greater than the Increased treatment of the current study.

The current study and that of Leonard et al. (2020), which utilized litter sizes close to current U.S. industry levels, suggested no benefit, in terms of pre-weaning piglet mortality and growth, from providing increases in farrowing pen sizes above those that are widely used in commercial practice in the United States. However, with continuing increases in litter sizes, further research in this area is warranted, including emphasis on understanding the effect of farrowing pen size on piglet behavior.
